# A fully human CXCR4 antibody demonstrates diagnostic utility and therapeutic efficacy in solid tumor xenografts

**DOI:** 10.18632/oncotarget.7111

**Published:** 2016-02-01

**Authors:** Babak Behnam Azad, Samit Chatterjee, Wojciech G. Lesniak, Ala Lisok, Mrudula Pullambhatla, Zaver M. Bhujwalla, Martin G. Pomper, Sridhar Nimmagadda

**Affiliations:** ^1^ The Russell H. Morgan Department of Radiology and Radiological Science, Johns Hopkins University, Baltimore, Maryland, USA

**Keywords:** chemokine receptor, Zirconium-89, cancer therapy, molecular imaging, precision medicine

## Abstract

For physiologically important cancer therapeutic targets, use of non-invasive imaging for therapeutic guidance and monitoring may improve outcomes for treated patients. The CXC chemokine receptor 4 (CXCR4) is overexpressed in many cancers including non-small cell lung cancer (NSCLC) and triple negative breast cancer (TNBC). CXCR4 overexpression contributes to tumor growth, progression and metastasis. There are several CXCR4-targeted therapeutic agents currently in clinical trials. Since CXCR4 is also crucial for normal biological functions, its prolonged inhibition could lead to unwanted toxicities. While CXCR4-targeted imaging agents and inhibitors have been reported and evaluated independently, there are currently no studies demonstrating CXCR4-targeted imaging for therapeutic guidance.

Monoclonal antibodies (mAbs) are commonly used for cancer therapy and imaging. Here, an ^89^Zr-labeled human CXCR4-mAb (^89^Zr-CXCR4-mAb) was evaluated for detection of CXCR4 expression with positron emission tomography (PET) while its native unmodified analogue was evaluated for therapy in relevant models of NSCLC and TNBC. *In vitro* and *in vivo* evaluation of ^89^Zr-CXCR4-mAb showed enhanced uptake in NSCLC xenografts with a high expression of CXCR4. It also had the ability to detect lymph node metastases in an experimental model of metastatic TNBC. Treatment of high and low CXCR4 expressing NSCLC and TNBC xenografts with CXCR4-mAb demonstrated a therapeutic response correlating with the expression of CXCR4. Considering the key role of CXCR4 in normal biological functions, our results suggest that combination of ^89^Zr-CXCR4-mAb-PET with non-radiolabeled mAb therapy may provide a precision medicine approach for selecting patients with tumors that are likely to be responsive to this treatment.

## INTRODUCTION

The CXC chemokine Receptor 4 (CXCR4), a G-protein coupled receptor, interacts with its endogenous ligand, CXCL12, to mediate normal biological functions including stem cell homeostasis. It also plays an important role in several other pathological processes including lupus, rheumatoid arthritis and as a co-receptor for the human immunodeficiency virus (HIV) [1, 2]. CXCR4 over expression in tumors contributes to growth, progression and metastasis [1, 2]. That overexpression in primary tumors is correlated with the degree of lymph node metastasis, increased risk of local recurrence and overall poor survival rates in a number of malignancies including non-small cell lung cancer (NSCLC) and breast cancers [3–8]. Several CXCR4 therapeutics are in clinical trials. The overexpression of CXCR4 in cancers and its importance in normal biological functions promotes the need for identification of tumors most likely to be responsive in order to achieve better therapeutic outcomes.

NSCLC is a leading cause of cancer fatality with metastatic spread accounting for >70% of patient mortality [9]. While oncogene-directed therapies have improved outcomes for 60% of NSCLC patients, chemotherapy remains the primary option for the remaining patient population [10]. A meta-analysis of 1446 NSCLC patients confirmed higher CXCR4 expression in NSCLC compared to normal lung tissue [11]. This overexpression was also associated with increased clinical stage, metastatic status and overall poor survival [12]. In other reports, elevated cytomembranous CXCR4 expression in NSCLC specimens was shown to correlate with an increased tendency for local invasion and distant metastases [13–15]. These studies suggest that CXCR4-targeted therapies may provide an applicable therapeutic approach for NSCLC.

Another cancer with a significant mortality rate is triple negative breast cancer (TNBC) that is negative for estrogen receptor (ER), progesterone receptor (PR) and human epidermal growth factor receptor-2 (Her-2). TNBC is associated with poor prognosis, a high level of local and distant recurrence and poor disease-free survival rates [16]. Conventional chemotherapy following surgical resection remains the primary approach to treatment [17, 18]. Nearly 75% of TNBCs exhibit high levels of activated CXCR4 and this activation leads to tumor growth and correlates with formation of distant metastases [19, 20]. CXCR4-targeted agents may prove to be effective therapeutics for treatment of primary and metastatic TNBC.

Targeting CXCR4 has shown therapeutic promise in preclinical models of various cancers [1]. Blocking the CXCR4/CXCL12 axis with low molecular weight (LMW) agents, peptides or antibodies has been shown to reduce tumor growth, metastasis and to sensitize cytotoxic chemotherapy in preclinical models [4, 7, 21, 22]. CXCR4 inhibitors are currently in clinical trials as anti-cancer therapeutics (*e.g.* ALX-0651 [NCT01374503], MSX-122 [NCT00591682], BMS-936564 [NCT02305563, NCT01359657, NCT01120457, NCT02472977]) [23]. The CXCR4 inhibitor Plerixafor was recently FDA approved for hematopoietic stem cell mobilization in patients with non-hodgkin lymphoma and multiple myelomas. CXCR4-targeted imaging agents have also been developed and a ^68^Ga-labeled CXCR4 binding peptide has shown promising results in lymphoproliferative disorders in patients [24–27]. Targets such as CXCR4 that play a critical role in normal physiological processes are likely to have a low therapeutic threshold. Although CXCR4 targeted therapeutics and imaging agents are in clinical trials, there are currently no studies on using CXCR4-targeted imaging for therapeutic guidance. In this study, we have attempted to establish a relationship between CXCR4 expression levels, CXCR4 targeted-imaging agent uptake and CXCR4-dependent therapeutic efficacy.

Monoclonal antibodies (mAbs) are gaining attention as therapeutics owing to their high antigen specificity, affinity and low off-target effects [28]. The fully human anti-hCXCR4 antibody MDX-1338 (CXCR4-mAb) has a high affinity for CXCR4 (EC50 = 2 nM for inhibition of ^125^I-CXCL12) and has shown promising therapeutic response in hematopoietic tumors but has not been evaluated in solid tumors [29]. Positron emission tomography (PET) using Zirconium-89 (t_1/2_ = 78.4h) as a radioactive label for an antibody has the utility for non-invasive *in vivo* detection of CXCR4 expression in tumors. Here we report the evaluation of ^89^Zr-labeled MDX-1338 (^89^Zr-CXCR4-mAb) for identifying tumors with high CXCR4 expression. Considering that the therapeutic efficacy of MDX-1338 has not been evaluated for treatment of solid tumors, we demonstrate the therapeutic response of this mAb in NSCLC and TNBC xenografts. Collectively, our results demonstrate that ^89^Zr-CXCR4-mAb uptake and therapeutic efficacy of CXCR4-mAb are correlated with levels of CXCR4 expression.

## RESULTS

### Generation of ^89^Zr-labeled CXCR4-mAb

**Figure 1:**
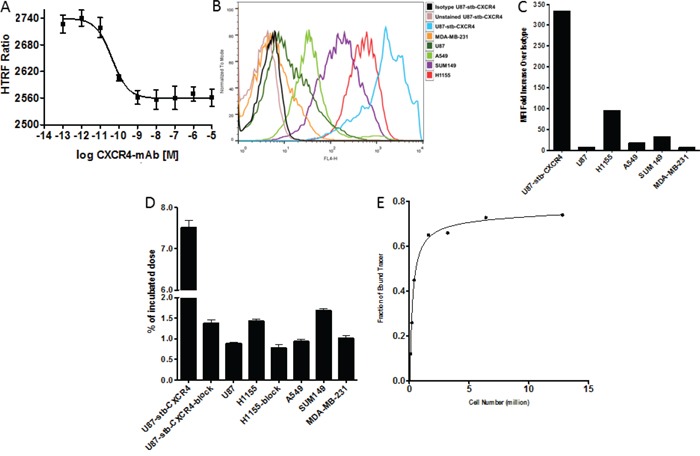
*In vitro* evaluation of CXCR4-mAb and 89Zr-CXCR4-mAb Representative *in vitro* competitive binding displacement assay of CXCR4-mAb against CXCL12-red **A.** Representative surface CXCR4 expression levels of studied cell lines analyzed by flow cytometry and illustrated as histograms **B.** and mean fluorescence intensity (MFI) **C.** *in vitro* binding specificity of ^89^Zr-CXCR4-mAb for graded levels of CXCR4 expression in various cells lines **D.** and an *in vitro* receptor saturation curve with ^89^Zr-CXCR4-mAb in U87-stb-CXCR4 cells **E.**

The half maximal inhibitory concentration (IC_50_) and inhibition constant (K_i_) of CXCR4-mAb for CXCL12-Red binding to CXCR4 were 43pM (95% confidence interval: 1.7 × 10^−11^ - 1.1 × 10^−10^) and 24pM (95% confidence interval: 9.6 × 10^−12^ - 6.1×10^−11^), respectively (Figure 1A). The control-mAb did not show CXCR4 affinity in the analyzed concentration range (10^−4^ to 10^−12^M).

Both CXCR4-mAb and the control-mAb were first conjugated with desferrioxamine (DFO) for ^89^Zr-chelation. Radiochemical yields for Zr-89 radiolabeling were 70 ± 5%. Antibody radiolabeling was confirmed with ITLC and autoradiography, resulting in radiochemical purities > 98% (n = 30). Specific activity values were 6.4±0.4 mCi/mg for *in vitro* studies and 2.5±0.1 mCi/mg for *in vivo* studies. SDS-PAGE (Coomassie staining) and autoradiography under reducing and non-reducing conditions indicated intact antibody after DFO conjugation and subsequent radiolabeling (data not shown).

### *In vitro* evaluation reflects a CXCR4-expression dependent ^89^Zr-CXCR4-mAb uptake

To evaluate the binding specificity of the ^89^Zr-CXCR4-mAb *in vitro*, uptake assays were carried out in glioblastoma (U87-stb-CXCR4, U87), NSCLC (H1155, A549) and TNBC (SUM149 and MDA-MB-231) cell lines. ^89^Zr-CXCR4-mAb showed increased binding in U87-stb-CXCR4, H1155 and SUM149 cell lines that correlated with the profile of CXCR4 expression observed by flow cytometry (Figure 1B-D). ^89^Zr-CXCR4-mAb uptake could be inhibited with 10meq of unlabeled CXCR4-mAb in U87-stb-CXCR4 and H1155 cell lines thus confirming a CXCR4-mediated binding. The immunoreactive fraction of the radiolabeled antibody, evaluated using reported Lindmo assays [30, 31], was 90±4% (Figure 1E).

### *In vivo* assessment demonstrates preferential ^89^Zr-CXCR4-mAb accumulation in NSCLC xenografts with high CXCR4 expression

**Figure 2:**
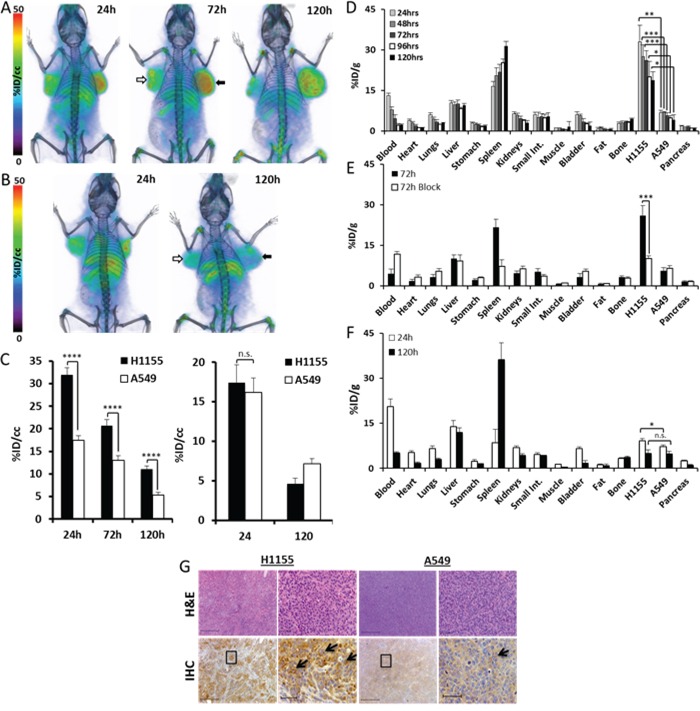
Analysis of 89Zr-CXCR4-mAb in H1155 and A549 NSCLC tumor xenograft models Volume rendered PET-CT images of mice harboring H1155 (black arrows) and A549 (white arrows) xenografts (n=5 per group or time point) injected with 250μCi of ^89^Zr-CXCR4-mAb **A.** or ^89^Zr-control-mAb **B.** Images acquired as well as image analysis for mice injected with ^89^Zr-CXCR4-mAb (**C**, left) or ^89^Zr-control-mAb (**C**, right) over 120h show CXCR4-specific radioactivity accumulation in CXCR4 high H1155 tumors; biodistribution studies in H1155/A549 xenografted mice over 120h show increased accumulation of radioactivity in H1155 tumors **D.** and when administered with a 10meq blocking dose prior to injection of ^89^Zr-CXCR4-mAb show a significant reduction in H1155 uptake (at 72h p.i.) **E.** ^89^Zr-control-mAb biodistribution in mice bearing H1155/A549 xenografts show no significant differences in radioactivity uptake between tumors at 120h p.i. **F.** H&E staining and CXCR4 immunohistochemistry (black arrows) of excised H1155 and A549 tumor tissues at 4x and 20x magnification (from black squares in 4x images) (scale 50μm) confirming higher CXCR4 expression in H1155 tumors **G.** p<0.05 *, <0.01 **, <0.001***, <0.0001****; n.s. denotes not statistically significant.

To evaluate the *in vivo* specificity of the radiolabeled antibody, we pursued imaging of NOD-SCID mice harboring high-CXCR4 H1155 and low-CXCR4 A549 xenografts. PET-CT imaging of these mice over 120h indicated preferential uptake of ^89^Zr-CXCR4-mAb in H1155 tumors compared to A549 tumors (Figure 2A). Attesting to the specificity of the ^89^Zr-CXCR4-mAb, no clear difference in uptake between tumors could be observed 120h after mice were injected with ^89^Zr-control-mAb (Figure 2B). Image analysis of mice injected with ^89^Zr-CXCR4-mAb (Figure 2C, left) or ^89^Zr-control-mAb (Figure 2C, right) further supported the imaging data showing CXCR4-mediated accumulation of the imaging agent in CXCR4-high tumors. Maximum imaging agent uptake values (%ID/cc) were 36.2±1.6 and 20.1±1.0 for ^89^Zr-CXCR4-mAb, compared to 23.6±2.2 and 20.7±1.8 %ID/cc for ^89^Zr-Control-mAb, in H1155 and A549 tumors respectively, 24hrs after imaging agent administration.

To further validate the imaging results, *ex vivo* biodistribution studies were carried out using ^89^Zr-CXCR4-mAb and ^89^Zr-control-mAb. These studies showed a consistently high accumulation of ^89^Zr-CXCR4-mAb in H1155 tumors with a %injected dose per gram (%ID/g) of 32.9 ± 6.2 at 24h and 21.6 ± 4.1 at 120h post injection (p.i.) (Figure 2D). In A549 tumors the %ID/g values were significantly lower at 6.7 ± 0.9 and 5.5 ± 1.1 at the same time points. H1155/A549 ratios were consistent at 4.3 ± 0.4 over the 120h study period. The highest tumor-to-blood (9.7 ± 2.2) and tumor-to-muscle (41.0 ± 9.7) ratios were observed at 96h p.i. Non-specific retention of radioactivity was observed in the liver and spleen at 25 and 8 %ID/g, respectively, at 96h p.i. These results were further confirmed by high CXCR4 immunoreactivity observed in excised H1155 tumors compared to A549 tumors (Figure 2G).

Confirming the CXCR4-mediated uptake, a significant reduction in %ID/g was observed in the H1155 tumors following the administration of a 10meq blocking dose of unmodified CXCR4-mAb (Figure 2E). The %ID/g values at 72 h p.i. were 10.2 ± 0.8 and 6.5 ± 1.1 for H1155 and A549 tumors, respectively. The target specificity was further confirmed by *ex vivo* biodistribution studies carried out with ^89^Zr-control-mAb at 24h and 120h p.i. time points, corresponding to highest tumor uptake and most clearance observed, respectively. Uptake of ^89^Zr-control-mAb was 4.9 ± 1.3%ID/g in H1155 and 4.8 ± 0.8%ID/g in A549 tumors, 120h p.i. (Figure 2F).

**Figure 3:**
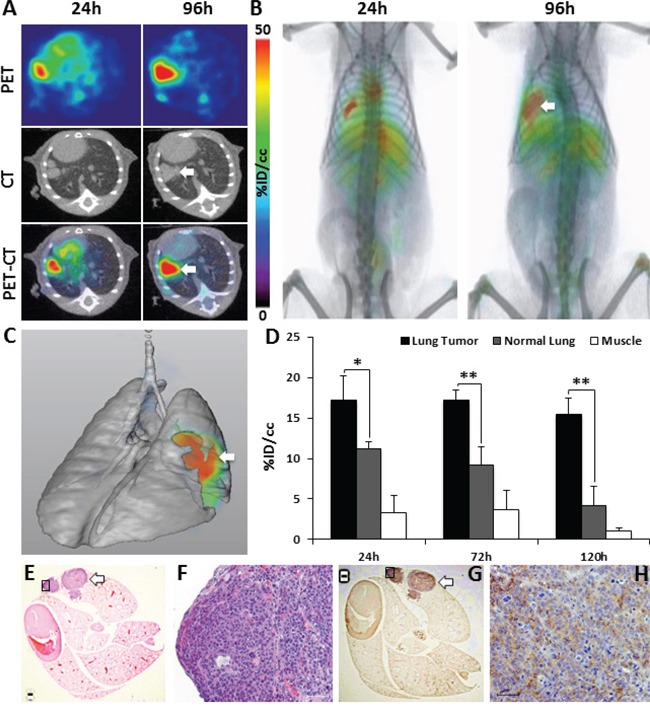
Evaluation of 89Zr-CXCR4-mAb in orthotopic H1155 tumor models PET-CT transaxial **A.** and volume rendered **B.** images of ^89^Zr-CXCR4-mAb in orthotopic H1155 mouse models (n=5) confirm tumor uptake (white arrow); volume rendered lung map illustrating the orthotopic H1155 tumor uptake of ^89^Zr-CXCR4-mAb (white arrow) **C.** PET image analysis of ^89^Zr-CXCR4-mAb uptake in H1155 lung tumors (black), normal lung (grey) and muscle (white) **D.** H&E staining **E, F.** and CXCR4 immunohistochemistry **G, H.** of excised lungs at 1.5x and 10x magnification (from black squares in 1.5 x images); scale 50μm; white arrows show CXCR4-expressing positive regions in the tumor tissue; p<0.05 *, <0.01 **.

Following the promising results observed in subcutaneous NSCLC xenografts, we tested the sensitivity of ^89^Zr-CXCR4-mAb for non-invasive *in vivo* detection of orthotopic NSCLC tumors. PET imaging of orthotopic H1155 tumor bearing mice showed clear uptake and retention of radioactivity in lung tumors, compared to contralateral lungs over the investigated 120 h time period (Figure 3A, 3B). Figure 3C is a representative volume rendered lung image, demonstrating the uptake of ^89^Zr-CXCR4-mAb in H1155 lung tumor. Image analysis indicated a tumor %ID/cc of 17.2 ± 3.1 and 15.5 ± 2.0, compared to 11.2 ± 0.4 and 4.2 ± 1.2 in contralateral lung, at 24h and 120h p.i., respectively (Figure 3D).

Hematoxylin and eosin (H&E) staining (Figure 3E, 3F) and CXCR4 immunohistochemistry of lungs (Figure 3. G, H) from the same mice further confirmed the presence of lung tumors and the CXCR4 expression within the tumors (black arrows). Collectively, above findings confirm the capability of ^89^Zr-CXCR4-mAb for non-invasive *in vivo* detection of CXCR4 expression in NSCLC xenografts.

### CXCR4-mAb treatment response in NSCLC xenografts correlates with ^89^Zr-CXCR4-mAb uptake and CXCR4 expression levels

**Figure 4:**
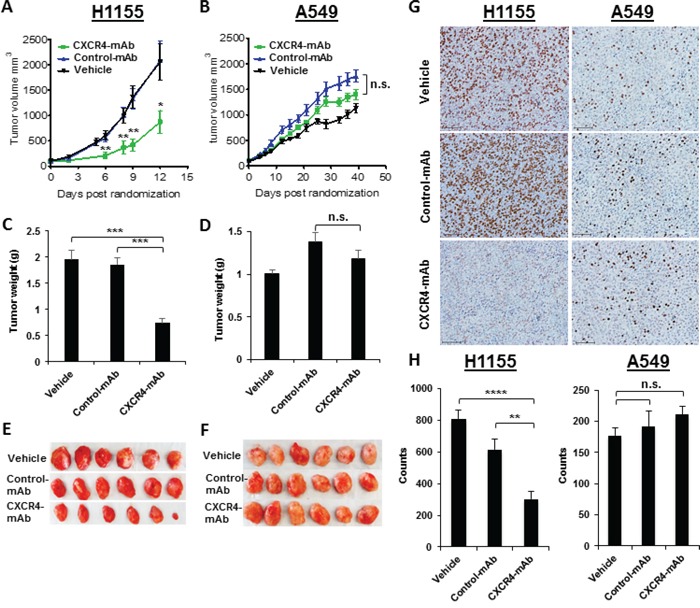
Therapeutic assessment of CXCR4-mAb in H1155 and A549 NSCLC tumor xenograft models Therapeutic studies (n=7 per cohort) with CXCR4-mAb, control-mAb or vehicle administered at a dose of 10 mg/kg every third day illustrate a significant reduction in tumor growth in high-CXCR4 H1155 tumors treated with CXCR4-mAb **A.** further confirmed by tumor weights post-therapy **C, E.** studies in low-CXCR4 A549 xenografts do not show clear therapeutic response **B.** also reflected in the final tumor weights **D, F.** BrdU staining (scale 50μm) of excised tumor tissues **G.** and quantification of the BrdU staining signal intensity **H.** showed a significant decrease in proliferation in high-CXCR4 H1155 tumors and no significant changes in proliferation for low-CXCR4 A549 tumors, further confirming that treatment response correlates with levels of CXCR4 expression; p<0.05 *, <0.01 **, <0.001***, <0.0001****; n.s. denotes not statistically significant.

We then investigated whether the imaging results observed with ^89^Zr-CXCR4-mAb could be used for therapeutic guidance. The therapeutic potential of CXCR4-mAb was evaluated in immunodeficient mice harboring H1155 and A549 NSCLC tumor xenografts. Mice were treated every third day with CXCR4-mAb, control-mAb or vehicle (saline), at a dose of 10 mg/kg, and tumor growth was measured. CXCR4-mAb treatment significantly reduced tumor growth in H1155 xenografts compared to controls (vehicle and control mAb) as early as 6 days following the start of treatment (Figure 4A). CXCR4-mAb treatment did not result in clear tumor growth reduction in A549 xenografts, even by the end of therapy (Figure 4B). These findings were also reflected in the final tumor weights (Figure 4C-F).

H1155 tumor weights from CXCR4-mAb treated mice were 60.0 ± 8.3% lower compared to the control-mAb and vehicle treated groups (Figure 4C). No significant differences in tumor weights were observed between the treatment groups in the A549 cohort (Figure 4D). In addition, reduced proliferation levels were observed in CXCR4-mAb treated H1155 tumors. BrdU staining of tumor sections showed significantly less proliferation in the CXCR4-mAb treated group compared to controls (Figure 4G, 4H). No significant differences in proliferation by BrdU staining were observed between treatment groups in A549 tumors. The observed agreement between PET-imaging and therapeutic response indicates the value of CXCR4-expression imaging in NSCLC for therapeutic guidance.

### ^89^Zr-CXCR4-mAb can detect TNBC lymph node metastases and CXCR4-mAb treatment response correlates with CXCR4 expression levels in TNBC xenografts

CXCR4 is highly expressed in TNBC and its expression correlates with poor patient prognosis [19, 20]. Large tissue microarray-based studies have also reported increased CXCR4 expression in metastases [32]. Because metastasis is still the primary cause for poor prognosis in patients with TNBC, we also assessed the potential of ^89^Zr-CXCR4-mAb for monitoring of lymph node involvement via CXCR4-targeting. An MDA-MB-231 cell line that spontaneously develops lymph node metastases with stably expressing luciferase (MDA-MB-231-Luc) was used for cross correlative studies.

**Figure 5:**
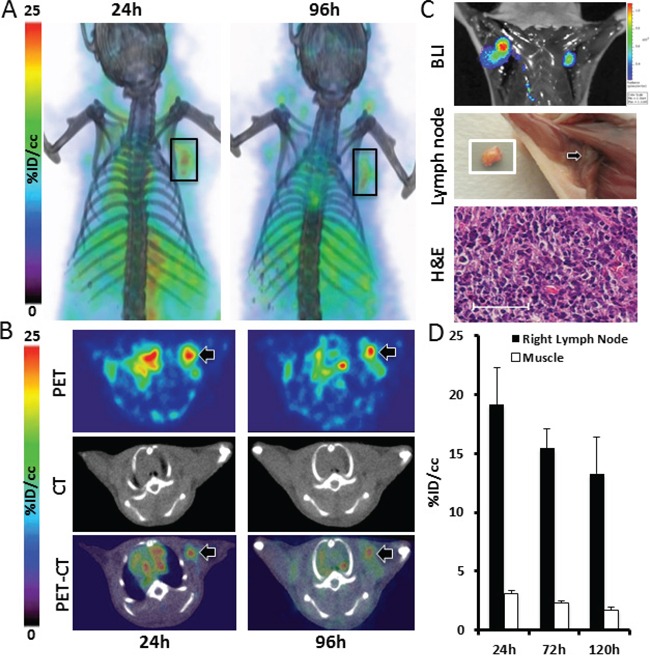
89Zr-CXCR4-mAb-PET for non-invasive detection of TNBC lymph node metastases Volume rendered **A.** and transaxial PET-CT **B.** and bioluminescence **C.** images of MDA-MB-231-luc derived metastatic mouse models (n=5) along with photographed image of excised lymph node (white square) and its H&E analysis (**C**) showing the applicability of ^89^Zr-CXCR4-mAb for *in vivo* detection of lymph node metastases (scale 50μm); image analysis of acquired PET images showing enhanced accumulation of ^89^Zr-CXCR4-mAb in lymph nodes (black bars) **D.**

PET imaging of mice harboring MDA-MB-231-Luc xenografts demonstrated ^89^Zr-CXCR4-mAb uptake by axillary lymph node metastases over 120h p.i. Figure 5A and B illustrate representative PET-CT images obtained at 24 and 96h post injection of ^89^Zr-CXCR4-mAb. Lymph node metastases were further confirmed by *in vivo* bioluminescence imaging and H&E staining of excised tissues (Figure 5C). PET image analysis indicated a %ID/cc of 19.1 ± 3.2 and 13.3 ± 3.1 in the right lymph node at 24h and 120h p.i., respectively (Figure 5D). These results demonstrate the applicability of ^89^Zr-CXCR4-mAb for detection of lymph node metastases in TNBC.

**Figure 6:**
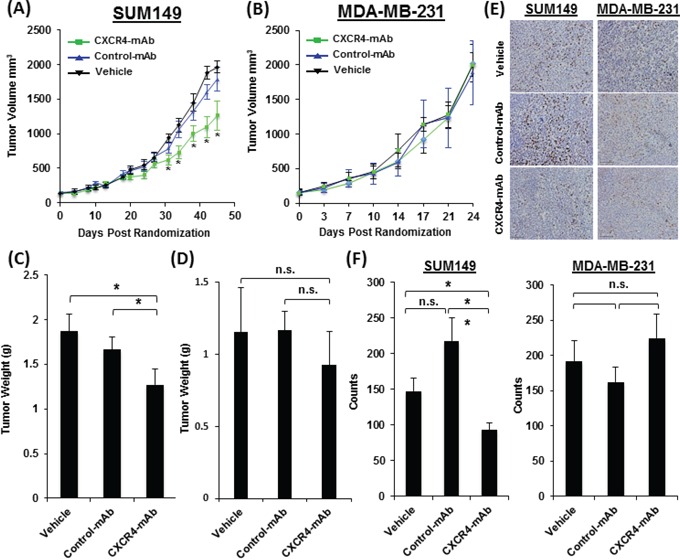
Therapeutic assessment of CXCR4-mAb in SUM149 and MDA-MB-231 TNBC tumor xenograft models Therapeutic studies with CXCR4-mAb, control-mAb or vehicle (n=7 per cohort) administered at a dose of 10 mg/kg every third day demonstrate a significant reduction in tumor growth in high-CXCR4 SUM149 tumors treated with CXCR4-mAb **A.** further confirmed by tumor weights post-therapy **C.** studies in low-CXCR4 MDA-MB-231 model do not show statistically significant differences in tumor growth between treatment groups **B.** also reflected in final tumor weights **D.** BrdU staining (scale 50μm) of excised tumor tissues **E.** and quantification of the BrdU staining signal intensity **F.** showed a significant decrease in proliferation in high-CXCR4 SUM149 tumors and no statistically significant changes in proliferation in low-CXCR4 MDA-MB-231 tumors further confirming that treatment response was correlated with levels of CXCR4 expression; p<0.05 *, <0.01 **; n.s. denotes not statistically significant.

To further validate the correlation between CXCR4 expression levels and treatment response observed in the NSCLC models, we chose TNBC, which has few targeted therapy options, and assessed whether increased CXCR4 expression correlated with improved therapeutic response in xenograft models of TNBC. Mice harboring SUM149 xenografts with a high expression of CXCR4 were treated with CXCR4-mAb and showed a significant reduction in tumor growth (Figure 6A) compared to control groups (vehicle and control-mAb). The same therapeutic regimen did not result in tumor growth reduction in low-CXCR4 MDA-MB-231 xenografts (Figure 6B). The reduced tumor growth in SUM149 was further reflected in final tumor weights. SUM149 tumor weights from CXCR4-mAb treated mice were 23.6±0.3% and 31.9±0.4% lower compared to control-mAb and vehicle groups, respectively (Figure 6C). No significant changes in tumor weights were observed in mice harboring low-CXCR4 expressing MDA-MB-231 tumors (Figure 6D). Proliferation levels, detected by BrdU staining of excised tumor sections were also lower in CXCR4-mAb treated SUM149 mice (Figure 6E, 6F). These results further confirmed a treatment response that is correlated with levels of CXCR4 expression and illustrate the potential of this CXCR4-mAb as a viable therapeutic option for the treatment of CXCR4 over expressing TNBC.

## DISCUSSION

Our studies using a human CXCR4-mAb show increased uptake of the imaging agent, ^89^Zr-CXCR4-mAb, in NSCLC xenografts with a high expression of CXCR4. These results demonstrate the ability of radiolabeled CXCR4 antibodies for non-invasive phenotyping of tumors for CXCR4 expression. In addition, a correlation between CXCR4 expression and therapeutic response was observed in NSCLC and TNBC tumor models, suggesting that CXCR4 imaging could be used for identification of tumors most responsive to CXCR4-targeted therapies.

Recently, the use of molecular imaging for greater personalization of therapy was shown to be effective in clinical regimens [33, 34]. The pivotal role of the CXCR4 receptor in tumor growth, metastasis and therapeutic resistance has been well documented [1, 2, 35]. Several CXCR4 inhibitors and imaging agents are in Phase I/II clinical trials [23]. Our studies with PET imaging of ^89^Zr-CXCR4-mAb in subcutaneous and orthotopic mouse models of NSCLC verified that the variable uptake of the imaging agent was based on CXCR4 expression levels. In all cases, tumors with high CXCR4 expression showed consistently enhanced uptake of ^89^Zr-CXCR4-mAb over 120h p.i., in comparison to tumors with low CXCR4 expression. The high tumor uptake and retention of ^89^Zr-CXCR4-mAb by CXCR4 overexpressing tumors make this imaging agent a viable tool for *in vivo* detection of CXCR4 expression. Tumor hypoxia could increase CXCR4 expression evident by high accumulation of ^89^Zr-CXCR4-mAb in the center of H1155 tumors. Similar results were observed with other CXCR4 antibodies [36]. The rapid growth of the H1155 tumors also resulted in reduced and more diffusive uptake in the same tumors by 120h. Factors such as rapid tumor growth, possible necrosis and reduced permeability could decrease the radiolabeled antibody uptake in the tumors. Our imaging and biodistribution studies, using whole tumors, show that uptake of ^89^Zr-CXCR4-mAb correlated with the levels of CXCR4 expression and therapeutic efficacy in the same tumor models. These results indicate the potential of ^89^Zr-CXCR4-mAb-PET for image-guided therapy of CXCR4-expressing NSCLCs. The non-specific uptake by liver and spleen may be attributed to the eventual capture/breakdown of the mAb by lymphocytes and hepatocytes [37]. The increase in bone uptake observed over 120h may be a result of transchelation and sequestration of Zr-89 to phosphate groups in bones [38]. While a number of imaging agents targeting the CXCR4 receptors have been reported (e.g. [^64^Cu]AMD3100, [^64^Cu]AMD3465, ^125^I-12G5), the use of radiolabeled CXCR4-mAb as a companion diagnostic for MDX-1338 may be more beneficial in obtaining information on antibody biodistribution as made evident in ^89^Zr-trastuzumab and bevacizumab studies in patients with breast cancer [39].

Since lymph node involvement at the initial diagnosis of breast cancer may be indicative of poor outcomes, [40] early detection of lymph node metastases may lead to more effective treatments and improved prognosis. Overexpression of CXCR4 in primary breast tumors is directly correlated to the degree of lymph node metastasis and poor survival rates in breast cancer patients, which suggest that CXCR4 expression could be used as a prognostic marker [41–43]. Our results in an experimental mouse model of metastatic TNBC demonstrate that ^89^Zr-CXCR4-mAb-PET can be used for detection of lymph node metastases. Our results also show a CXCR4-expression dependent therapeutic effect of the CXCR4-mAb for reducing tumor growth in TNBC xenografts.

In addition to TNBC, CXCR4 overexpression also occurs in other breast cancer subtypes [7, 20, 42]. HER-2-negative breast tumors with an overexpression of CXCR4 demonstrate more aggressive behavior and are more likely to recur than tumors that do not express elevated levels of CXCR4 [44]. Similarly, CXCR4 has been shown to mediate estrogen-independent tumorigenesis, metastasis and resistance to endocrine therapy. CXCR4 overexpression is correlated with worse prognosis and decreased patient survival irrespective of ER status [5]. Non-invasive imaging of ER with [^18^F]fluoroestradiol ([^18^F]FES-PET) has been shown to be predictive of response to endocrine treatment in breast cancers [45, 46]. Similarly, demonstration of HER-2 expression by nuclear imaging techniques is under study [47]. However, these agents are not suitable for TNBCs. Because CXCR4 is expressed in all subtypes and to the highest degree in TNBCs [20], breast cancer patients who do not benefit from hormonal or anti-HER-2 therapy may potentially benefit from CXCR4 targeted therapies. Our results in TNBC models could therefore be applied in other subtypes of breast cancers.

The fully human CXCR4 mAb, MDX-1338, was recently shown to effectively block calcium flux and migration of cancer cells, induce apoptosis in various tumor cell lines *in vitro* and reduce tumor growth in xenograft models of acute myeloid leukemia, non-Hodgkin lymphoma and multiple myeloma [29]. MDX-1338 is currently in phase I clinical trials for treatment of relapsed and refractory AML, NHL, chronic lymphoid leukemia and MM (NCT01120457). Similarly, a CXCR4 targeted nanobody and several low molecular weight agents and peptide derivatives are in clinical trials [23]. Results obtained from our study suggest that CXCR4-targeted imaging will aid in the development of therapeutic regimens based on the status of tumor CXCR4 expression and towards realizing precision medicine. Additionally, radiolabeled antibody accumulation in the tumors can be correlated with the degree of therapy response. Such imaging driven studies would encompass and integrate the tumor heterogeneity and physiology in therapy planning, as we have attempted to demonstrate in the present study.

CXCR4 targeted antibodies could also have certain advantages as therapeutics. Although chemotherapeutics such as gemcitabine induce CXCR4 expression, combination of CXCR4 inhibitors and chemotherapeutics have shown synergistic therapeutic effects [1, 23]. Use of CXCR4 antibodies in combinatory therapeutic approaches may be beneficial owing to fewer off target effects.

In summary, we have developed and evaluated the imaging and therapeutic potential of a CXCR4-mAb in solid tumor models. Our data shows that ^89^Zr-CXCR4-mAb is able to identify CXCR4 overexpressing tumors and metastases. Therapeutic evaluation of the CXCR4-mAb in NSCLC and TNBC xenografts revealed that tumors with higher CXCR4 expression are more responsive to CXCR4-targeted therapy. Our data shows the importance of CXCR4-targeted imaging for therapeutic guidance and provides a rationale for selecting highly CXCR4-positive tumors for CXCR4-inhibition for improved therapeutic outcomes.

## MATERIALS AND METHODS

All reagents were purchased from Sigma Aldrich (St. Louis, MO) unless otherwise specified. DFO was purchased from Macrocyclics (Dallas, TX). Spin columns were purchased from EMD Millipore (Billerica, MA) and TLB buffer was purchased from Cisbio. The fully human CXCR4-mAb was custom produced by Evitria AG (Zurich, CH) with cloning of the corresponding cDNAs (HC & LC) into Evitria's vector system using conventional (non-PCR-based) techniques. The gene fragments as well as Evitria's vector plasmids were gene synthesized. Plasmid DNA was prepared under low-endotoxin conditions using commercially available DNA purification kits. CHO-K1 cells were used for mAb production. The seed was grown in eviGrow media, a chemically defined, animal-component free, serum-free media. Transfection and production was made in eviMake at 37°C and 5% CO_2_. The supernatant was harvested by centrifugation and sterilely filtered (0.2 μm) 8 days after transfection. The antibody was purified based on Protein A affinity chromatography and rebuffered into PBS. Isotype matched IgG4 antibody (Pallivizumab, control-mAb), raised against respiratory syncytial virus, was used as control.

### *In vitro* affinity

Half maximal effective concentrations (IC50) were determined as previously reported by our group using a frequency resonance energy transfer-based multi-concentration competitive binding assay in CHO1-CXCR4-SNAP-Lumi4-Tb cells (Cisbio Bioassays) [48]. Briefly, 1×10^4^ cells in 10μL TLB buffer per well (in 384-well plates and done in triplicates) were mixed with 5μL of 60 nM of fluorochrome conjugated CXCL12 (CXCL12-Red) in TLB buffer and 5μL of TLB buffer containing increasing concentrations of the inhibitors ranging from 4×10^−5^M to 4×10^−13^M. Following a 2h incubation at room temperature, a Homogeneous Time-Resolved Fluorescence (HTRF) analysis with excitation at 340 nm and emissions at 620 nm and 665 nm (delay 50μs, window time 400μs, measurement time 1s) was performed using a Perkin Elmer Victor^3^V 1420 multi-label counter. All calculations were done using GraphPad Prism 6 software (GraphPad Software, Inc., San Diego, CA).

### Synthesis of the mAb-DFO conjugates

In a typical reaction, 0.5 mg of mAb in 200μL of saline was adjusted to pH 8.9-9.1 with 0.1M Na_2_CO_3_. To this solution a 5 fold excess of DFO in DMSO was added, ensuring less than 2% (v/v) DMSO in the final solution, followed by incubation at 37°C for 45 min. The resulting mAb-DFO was purified by size exclusion chromatography using Amicon Ultracel® 10K centrifugal filters (Merck Millipore Ltd., Tullagreen, Carrigtwohill, Co. Cork, IRL). The final concentration of the mAb-DFO conjugate was determined using a ND-1000 Nanodrop spectrophotometer (Thermo Fisher Scientific, Wilmington, DE, USA).

### Radiolabeling with Zirconium-89

In a typical reaction, ^89^Zr-oxalate was neutralized with 2M Na_2_CO_3_ to pH 7. This was followed by addition of 0.5 mg of the mAb-DFO conjugate in 0.5mL of 0.5M HEPES buffer (pH 7.1-7.3) and incubation at RT for 1 h with gentle shaking. Radiolabeling was monitored by ITLC using a pH 4.9-5.1 citric acid buffer with an R_f_ value of 0.0-0.1 for the radiolabeled antibody and an R_f_ > 0.1 for unbound radioactivity. Radiochemical yields were 65±5% with radiochemical purities > 98%. ^89^Zr-DFO-mAb was purified and concentration was measured as described previously.

Antibodies were electrophoretically ran in 1mm NuPAGE Novex Gel, either under reducing (sample with 0.7M 2-mercaptoethanol, heated at 95°C for 2 min before loading) or non-reducing conditions (without 2-mercaptoethanol) and were stained with colloidal Coomassie G-250 as per the manufacturer's protocol (SimplyBlue^TM^ - Life Technologies). The stained gel was then exposed to X-ray film overnight for autoradiography.

### Cell lines

All cell culture reagents were purchased from Invitrogen unless otherwise noted. Human NSCLC (H1155, A549), primary glioblastoma (U87) and breast cancer (MDA-MB-231) cell lines were purchased from the American Type Culture Collection (ATCC) and cultured in our laboratory. A U87 cell line stably transfected with human CXCR4 (U87-stb-CXCR4) was obtained from NIH AIDS Research and Reference Reagent Program (Dr. Hong Kui Deng and Dr. Dan R. Littman) and maintained in DMEM supplemented with 15% FBS, 1μg/mL puromycin, 300μg/mL G418, 100units/mL of penicillin, and 100 mg/mL of streptomycin. MDA-MB-231 expressing luciferase (MDA-MB-231-Luc) and SUM149 (Brest cancer) cells were kind gifts from Dr. Aleksander Popel of JHU and Dr. Steve Ethier of Medical University of South Carolina, respectively. MDA-MB-231 and MDA-MB-231-Luc Cell lines were maintained in RPMI-1640 with 10% fetal bovine serum (FBS) and 1% penicillin-streptomycin (P/S). SUM149 cells were maintained in Ham's F-12 with 5% FBS, 1% P/S and 250μL of 10 mg/mL insulin. A549 cells were maintained in F-12K with 10% FBS and 1% P/S. H1155 cells were maintained in RPMI 1640 with 5% FBS and 1% P/S. All cell lines were maintained in a humidified incubator under 5% CO_2_ at 37°C.

### Flow cytometry

Cells at 50-70% confluency were detached using a non-enzymatic cocktail (Gibco) and washed twice with flow cytometry buffer (1XPBS, 2mmol/L EDTA, 0.5% FBS). CXCR4 expression was determined by immunostaining with the allophycoerythrin (APC)-conjugated anti-human CXCR4 antibody (clone12G5, R&D Systems) according to the manufacturer's instructions. CXCR4 expression was analyzed on a FACSCalibur flow cytometer (Becton Dickinson). Data analysis was carried out using FlowJo software.

### *In vitro* binding assays

*In vitro* binding assays were carried out, at least in triplicate, and repeated three times in the respective growth media over one hour at 37°C using one millions cells. Blocking studies were carried out using a 10meq excess of the unmodified CXCR4-mAb. Following incubation, cells were rinsed with cold PBS three times and pellets were counted in an automated gamma counter (1282 Compugamma CS, Pharmacia/LKBNuclear, Inc., Gaithersburg, MD). Immunoreactive fractions were determined based on established literature reports [30, 31].

### Mouse xenografts

All animal studies were carried out according to regulations set forth by the Johns Hopkins Animal Care and Use Committee. Female, 6-8 weeks old, Non-Obese Diabetic Severe-Combined Immunodeficient (NOD-SCID) mice were purchased from Johns Hopkins immune compromised animal core. Mice were inoculated with one million H1155, A549, SUM149 or MDA-MB-231 cells in 100mL of HBSS in the upper flanks. Mice bearing tumors of 4-6mm in diameter were utilized for *in vivo* PET-CT imaging or *ex vivo* biodistribution studies.

Orthotopic mouse models bearing lung tumors were developed according to published procedures [49]. Briefly, mice were anesthetized and placed in a right lateral position. A 1cm skin incision was made on the scapula followed by separation of muscles to expose the costal layer. Approximately 1×10^6^ H1155 cells in 30μL of the culture medium were directly injected through the intercostal space into the left lobe of the lung using a 29G needle syringe. Skin incisions were closed with polypropylene sutures and the mice monitored under a heat lamp until full recovery. Orthotopic lung tumor development was monitored by Computed Tomography (CT) imaging.

The MDA-MB-231-Luc cell line was used to generate a spontaneous metastatic mouse model. For this model, athymic nude mice were inoculated with one million MDA-MB-231-Luc cells in 100mL of HBSS in the lower mammary fat pads. Lymph node metastases were confirmed by bioluminescence imaging acquired using a Xenogen® IVIS Spectrum imaging system (Xenogen Corporation, Alameda, California, USA). Mice were injected with 100 mg/kg of Luciferin i.p, anesthetized with 3% isoflurane and maintained at 1% isoflurane prior to being placed on the scanner bed. Exposures times ranged from 1-3min. Bioluminescence images were acquired pre and post sacrifice for visualization of lymph node metastases and correlated with PET-CT images obtained with ^89^Zr-CXCR4-mAb.

### PET-CT imaging of mouse xenografts

Mice were injected intravenously with 250mCi of ^89^Zr-CXCR4-mAb or ^89^Zr-control-mAb in 200mL of saline (n = 5), anesthetized with 3% isoflurane, prior to being placed on the scanner bed, and kept warm with an external light source while being scanned. Mice were maintained at 1% isoflurane during imaging. PET imaging (2 beds; 15mins per bed) was carried out using an eXplore VISTA small-animal PET scanner (GE Healthcare Life Sciences, Pittsburgh, PA, USA). A CT scan (512 projections) was performed at the end of each PET scan for anatomical co-registration using a CT-equipped Gamma Medica-Ideas SPECT scanner (Northridge, CA). PET data were reconstructed using the three-dimensional ordered subsets-expectation maximization algorithm (3D-OSEM) and corrected for dead time and radioactive decay. The %ID per cc was calculated based on a calibration factor obtained from a known quantity of radioactivity. Final visualization data and image generation was accomplished using Amira® (FEI, Hillsboro, OR). Quantification of PET images was carried out in AMIDE medical imaging data examiner using geometric region of interest (ROI)-generated volume statistics.

### Biodistribution studies

Mice bearing either lung or breast xenografts were injected intravenously with 35mCi of ^89^Zr-CXCR4-mAb or ^89^Zr-control-mAb in 100mL of saline. Mice (n = 4 per time point) were sacrificed at 24, 48, 72, 96 and 120h post injection. For blocking experiments, mice were injected intravenously with 1 mg of the unmodified CXCR4-mAb 1h prior to injection of ^89^Zr-CXCR4-mAb. Blood, liver, spleen, heart, lungs, kidneys, small intestines, large intestines, stomach, muscle, fat, bone, bladder and tumors were retrieved. Each sample was weighed and counted using an automated gamma counter (1282 Compugamma CS, Pharmacia/LKBNuclear, Inc., Gaithersburg, MD). The percentage of injected dose per gram of tissue (%ID/g) was then calculated, accounting for decay correction, using external Zr-89 standards, which were measured in triplicate.

### Immunohistochemistry

Retrieved subcutaneous tumors, tumor bearing lungs and lymph nodes were evaluated by H&E staining and for CXCR4 by immunohistochemistry (IHC). Harvested tissues were fixed in 10% buffered formalin and embedded in paraffin prior to sectioning with a 4μm thickness. After deparaffinizing, tumor sections were treated with 3% H_2_O_2_ (DAKO, Carpinteria, CA) for 10min and then incubated with a primary anti-CXCR4 antibody (clone UMB-2, Abcam) with a 1:50 dilution for 20min in a humidified chamber at room temperature. Slides were subsequently washed and incubated with secondary anti-rabbit polymer antibody (DAKO) for 15min at room temperature. DAB staining was carried out according to the manufacturer's protocols. Sections were counterstained with Gill's Hematoxylin followed by dehydration with gradient alcohol and xylene washes prior to mounting with a cover slip.

Cell proliferation levels were assessed using BrdU staining. For this study, mice were injected with 1 mg of BrdU intraperitoneally 1h prior to euthanasia. Following deparaffinization, tissues were treated with peroxidase block (Dako) for 10min, 0.02% pepsin in 0.01N HCl for 15min at 37°C, 2N HCl for 30min, 0.1M pH 8.5 sodium borate buffer for 10min and 10% FBS for 30min with PBS washes after each step. Slides were then incubated with mouse anti-BrdU antibody (BD, clone B44, 1:100 dilution in antibody diluent) for 3h followed by treatment with anti-mouse rabbit IgG post primary and polymer secondary antibodies sequentially with PBS washes after each step. DAB staining was carried out according to the manufacturer protocols. Sections were counterstained with Gill's Hematoxylin followed by dehydration with gradient alcohol and xylene washes prior to mounting with a cover slip. Quantification of stained slides was carried out using ImageJ image processing and analysis software. Images were converted to 8-bit grayscale and analyzed using a particle size (pixel^2^) of 30-infinity and Circularity of 0.00-1.00.

### Therapeutic studies in NSCLC and TNBC xenografts

Mice with H1155, A549, MDA-MB-231 or SUM149 tumors were randomized when tumors reached ∼150mm^3^ and treated with vehicle (saline), CXCR4-mAb or control-mAb. CXCR4-mAb or Control-mAb was injected intraperitoneally (10 mg/kg every third day). Tumor volumes were measured with a digital caliper twice a week and calculated as (Length*(Width^2^))/2. The weight of the mice was monitored throughout the study (n=7 per treatment group).

### Statistical analysis

Statistical analysis of data from *in vitro* studies was carried out with GraphPad Prism 6.0 software using an unpaired two-tailed t-test with statistical significance set when p <0.05. Excel 2013 was used for statistical analysis of *in vivo* PET images, *ex vivo* biodistribution, as well as therapeutic response and tumor volume calculations. In all cases, an unpaired two-tailed t-test was used for determination of statistical significance set when p<0.05.
